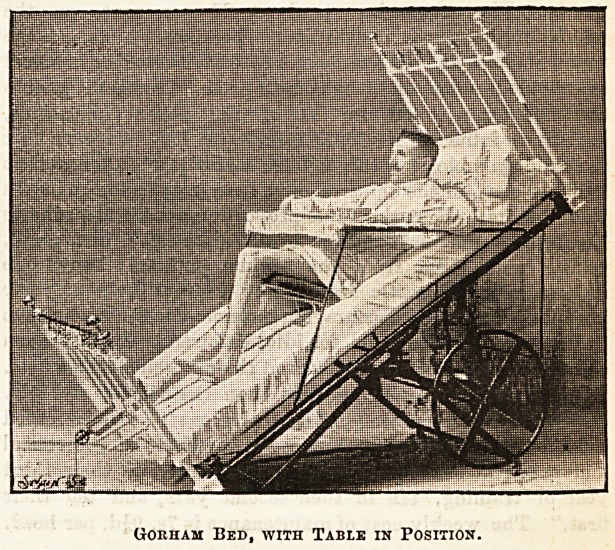# The "Gorham" Bed

**Published:** 1896-01-18

**Authors:** 


### The Institutional Workshop.

### PRACTICAL DEPARTMENTS.

### THE "GORHAM" BED.

Amongst the many improvements which recent years have seen in the shape and general construction of beds for sick people, this latest invention of an American physician, Dr. George E. Gorham, must take a high place. It is really a marvel of clever contriving, and in the case of bed-ridden patients a comfort, the greatness of which can perhaps be only properly estimated by themselves.

The chief purpose in view by the inventor has been the obviating of any unnecessary lifting and moving of a patient, and the giving easy changes of position without jar or discomfort. And the mechanism is such that every change in the position of the bed can be effected with very little exertion on the part of nurse or attendant. It is entirely made of iron and brass, and all movements are regulated by the one wheel under the bed which is seen in the illustration; either the head or foot can be raised by gradations to any height desired, and by an arrangement of straps and buckles a hammock-seat can be placed beneath the patient and with a bed tilted at a convenient angle, and the adjustment of a foot-rest, a very comfortable, almost sitting, position is effected.

But this by no means exhausts the possibilities of the Gorham bed. When the patient is thus reclining in a sitting posture a table can be slung in front which will carry dinner tray, writing materials, and even in one case a typewriter has been used upon it quite easily. As seen in the illustration, the "lift hammock" is also a great feature, for by this it is possible to raise the patient some ten inches above the mattress, and make the bed with satisfaction to the nurse and an entire absence of worry to the invalid. This hammock arrangement is also valuable in many other ways, facilitating dressings in surgical cases, and useful in cases of rheumatism or typhoid, and it can also be detached from the bed and used as a stretcher. Both the head and foot pieces can be entirely removed if the bed is to be used for an operation, for which purpose it really makes a perfect table; the height is thirty inches from the floor.

It is almost needless to say that wherever introduced the Gorham bed has been welcomed by doctors and nurses.

Gorham Bed, showing "Lift Hammock."

Several of the large London hospitals have adopted one or more in their wards; at the London, at St. Thomas's, the Middlesex, and others it is being found the greatest comfort in particular cases. Considering its construction and the many devices employed for the comfort of the occupant, and also that it is a thoroughly well made piece of furniture, the price is most moderate, £15 complete, "the very best,"

Gorham Bed, with Table in Position.

while in cheaper forms it is made for £9 and £5. We would suggest to charitably disposed people with a little money to spare that the presentation of one or more such beds to the wards of a hospital would be an excellent and most acceptable way of spending it. It would mean much alleviation of suffering, which would otherwise be unattainable, for few hospitals will be able to afford anything but a limited purchase of this most desirable luxury. Inquiries should be addressed to the "Gorham" Invalid Bed Company, 14A, Finsbury Square, E.C.

## Figures and Tables

**Figure f1:**
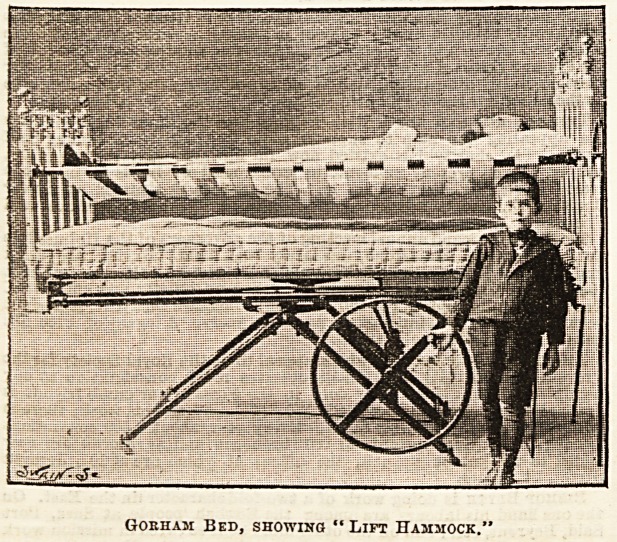


**Figure f2:**